# Critically coupled Fabry–Perot cavity with high signal contrast for refractive index sensing

**DOI:** 10.1038/s41598-021-98654-w

**Published:** 2021-10-01

**Authors:** Gyeong Cheol Park, Kwangwook Park

**Affiliations:** 1 Electronics and Telecommunications Research Institute, Daejeon, 34129 Republic of Korea; 2 Division of Advanced Materials Engineering, Jeonbuk National University, Jeonju, 54896 Republic of Korea; 3 Hydrogen and Fuel Cell Research Center, Jeonbuk National University, Jeonju, 54896 Republic of Korea; 4 Department of Energy Storage/Conversion Engineering of Graduate School, Jeonbuk National University, Jeonju, 54896 Republic of Korea

**Keywords:** Engineering, Optics and photonics, Physics

## Abstract

Perfect absorption at a resonance wavelength and extremely low absorption at the wavelength range of off-resonance in a one-port optical cavity is required for refractive index (RI) sensing with high signal contrast. Here, we propose and analyze an absorption-enhanced Fabry–Perot (MAFP) cavity based on a critical coupling condition in a near-infrared wavelength range. For a one-port cavity, a thick bottom Au is used as a mirror and an absorber. To achieve the critical coupling condition, a top dielectric metasurface is employed and tailored to balance the radiation coupling and the absorption coupling rates, and the one-port cavity is theoretically analyzed using temporal coupled-mode theory. We investigate two types of MAFP structures for gas and liquid. The gas MAFP cavity shows a sensitivity of ~ 1388 nm/RIU and a full-width at half-maximum of less than 0.7 nm. This MAFP cavity resolves the RI change of 5 × 10^−4^ with a reflectance signal margin of 50% and achieves a signal contrast of ~ 100%. The liquid MAFP cavity shows a sensitivity of ~ 996 nm/RIU when RI of liquid changes from 1.30 to 1.38. With tailoring the period of the metasurface maintaining its thickness, a signal contrast of ~ 100% is achieved for each specific RI range.

## Introduction

Sensing technology has been developed in various areas to diversify the limited scope and sensitivity of human senses. Among the variety of sensors, optical refractive index (RI) sensors have been widely used in bioanalytics, medical diagnostics, environmental monitoring, and material identification, etc.^1–3^. To precisely distinguish and exactly analyze various gases and liquids, there have been diverse optical resonance structures used such as Fabry–Perot (FP) interferometers, guide-mode resonance (GMR) structures, surface plasmon polariton (SPP) resonance structures, Tamm plasmon (TP) resonance structures, Fano resonance structures, and bound states in the continuum (BIC) structures, etc.^4–27^. Based on these resonance structures, the optical RI sensors detect the shift in a resonance peak or dip position (*λ*_0_) along the optical spectrum by reading a spectrometer before and after an analyte being covered. In other words, measuring the shift in the resonant wavelength (∆*λ*_0_), i.e., the change in the refractive index (∆n) due to the analyte is determined. To achieve a highly accurate optical RI sensor, ∆*λ*_0_ should be large enough to resolve and to measure the signal with high sensitivity. The performance of the optical RI sensors can, therefore, be assessed by measuring sensitivity defined as S = ∆*λ*_0_*/*∆n (nm/RIU, RIU: refractive index unit). To enhance the sensitivity, for one example, the cavity thickness or optical path length of a resonance structure to be filled or covered with an analyte should be long enough to distinguish the shift in a resonance wavelength^22,23^.

For precise sensing of an analyte, the quality factor (Q) of a resonance structure should be considered at the peak or dip. The Q-factor is defined as Q = ω_0_/∆ω, where ω_0_ is the resonance angular frequency and ∆ω is the full-width at half-maximum (FWHM). Accordingly, the resonance structure with a high Q-factor at ω_0_ possesses a narrow FWHM^24^. At the resonance, the Q-factor is another important parameter, which can improve the limit of detection (LOD) i.e., LOD = λ_0_/QS by reducing the overlap with the neighboring spectrum before an analyte being covered. With the same resonance wavelength shift, if the FWHM of a resonance structure becomes narrower, then it can achieve a larger LOD. To reduce the FWHM of a resonance structure, a dielectric metasurface is employed because it is lossless, and it excites distinctive guided-mode resonance depending on the surrounding analyte^17^. In addition, a dielectric metasurface can be designed having a high Q-factor and a narrow FWHM^19^. Other resonance structures such as Fano resonances structures and BIC structures have been proposed to further increase the Q-factor and hence to reduce the FWHM^12,13^.

Even though an optical resonance structure has a high Q-factor and a narrow FWHM, if a dip or peak can barely be distinguished from its background signal, it is difficult to determine the shift of the dip or peak in a resonance wavelength. Therefore, with a high Q-factor, an optical resonance structure should have a distinctive peak or dip from the background signal to be immune to noise. With this noise-immune resonance structure, the effect of the background noise can be further eliminated with deep learning^28^.

In this regard, the signal contrast (SC) is defined as the absolute difference between a dip or peak level (SIG_dip/peak_) and its background level (SIG_bg_), i.e., SC =|SIG_dip/peak_–SIG_bg_|. To clearly distinguish the dip or peak position from its background level, the SC of a resonance structure should be large as much as possible. The ideal SC is unity or 100% within a measured wavelength range. To achieve perfect absorption at a resonance wavelength, a resonance structure should be designed to balance a radiation coupling rate (*γ*_rad_) and an absorption coupling rate (*γ*_abs_). At the same time, in a condition of the off-resonance, the reflectance should approach unity or 100%. The absorption coupling rate can be tuned by controlling the thickness of a chosen absorbing material. In addition, if the absorbing material such as Au or Ag is used as a bottom mirror, the reflectance of these mirrors should be high enough to prevent the unwanted radiation coupling to an exit region. Having such a high reflective bottom mirror, a top mirror becomes the only path to control the radiation coupling rate to achieve the critical coupling condition. A dielectric grating mirror or a dielectric metasurface is a single-layer mirror and its reflectance can be controlled by changing its design parameters such as a period (Λ) and high-index grating bar width (*w*) but by keeping a grating thickness (*t*_g_)^29,30^. Besides, it can be used to tune the phase of FP cavities by tailoring the design parameters of an upper metasurface for the applications of spatial light modulation^25,26^. On the contrary, in the case of a distributed Bragg reflector (DBR), the reflectance can be coarsely tuned by changing the number of multiple DBR pairs, with more possibly only by changing the thickness of the uppermost DBR layer^31^. It is worthy to mention that diverse metamaterial absorbers including actively tunable meta-structure have been reported to achieve perfect absorption in the microwave and terahertz region^32–36^.

In this paper, we propose and analyze an absorption enhanced Fabry–Perot cavity for RI sensing based on the critical coupling condition, operating in a near-infrared (NIR) wavelength range. This cavity consists of a top dielectric metasurface and a bottom thick Au mirror, and hence forms a metasurface-Au Fabry–Perot (MAFP) cavity. To understand the absorption property of MAFP cavities depending on the design parameters of a dielectric metasurface, the temporal coupled-mode theory (TCMT) is used. Based on the understanding of the effect of the top dielectric metasurface on the coupling condition, two different types of a MAFP cavity for gaseous and liquid analytes sensing are investigated. The gas MAFP cavity shows an S of ~ 1388 nm/RIU and an FWHM of less than 0.7 nm. To achieve the high S, the top metasurface possesses air slits. The air slits pass gas into a cavity and maximize the effect of a RI change in the sensor. Moreover, the gas MAFP resolves the RI change of 5 × 10^−4^ with a reflectance signal margin of 50%, and an SC is ~ 100%. The liquid MAFP sensor shows an S of ~ 996 nm/RIU when the RI of liquid changes from 1.30 to 1.38. By tailoring the period of the metasurface but keeping its thickness, an SC of ~ 100% is achieved for each specific RI range.

## Concept and design

**Figure 1:**
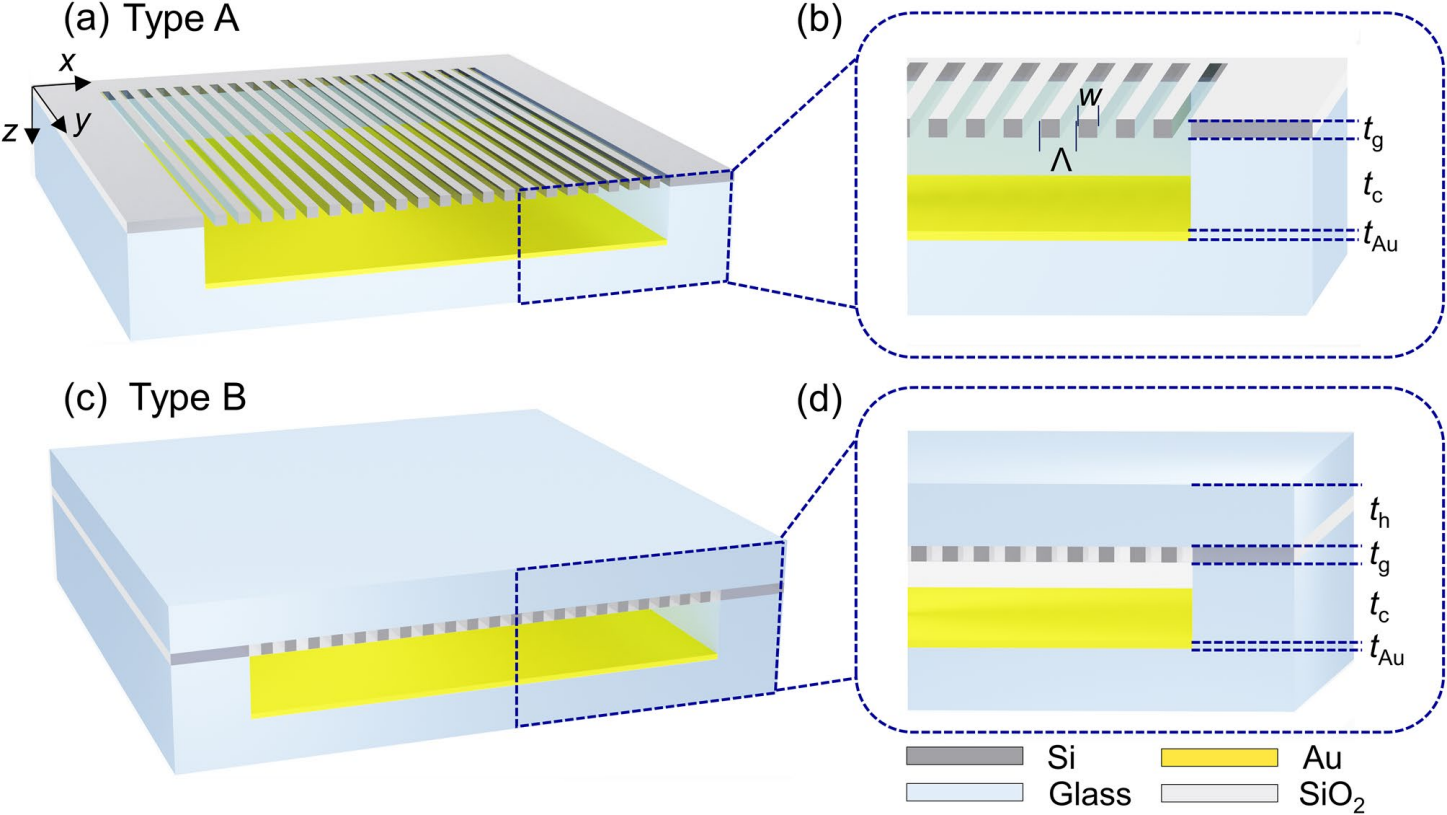
Schematic illustration of an absorption enhanced resonant structure (**a**) with an open-slit top dielectric metasurface and (**c**) with an enclosed dielectric metasurface by a glass handle. The two different resonant structures with their own distinct top dielectric metasurface are (a) for gas sensing and (**c**) for liquid sensing. The zoom-in cross-sectional view of each sensor is shown as (**b**) and (**d**), with the following design parameters: period (Λ), Si grating bar width (*w*), Si grating thickness (*t*_g_), cavity thickness (*t*_c_), Au thickness (*t*_Au_), glass handle thickness (*t*_h_).

Figure 1 shows the schematic configurations and its cross-sectional views of absorption enhanced resonance structures using a top dielectric metasurface and a bottom Au mirror separated by a spacer. The two different mirrors separated by the spacer form a metasurface-Au Fabry–Perot (MAFP) cavity. The top dielectric metasurface consists of 1D grating structures in which a low-index grating bar and a high-index grating bar are put next to each other, and presents a high index contrast^30,31,37^. The dielectric metasurface works as a high reflective mirror for transverse-electric (TE) polarization^37–39^. Unlike DBRs, the dielectric metasurface just requires a single-layered structure to achieve nearly 100% reflectance. Since it is a dielectric, there is no loss in the top metasurface, and the reflectance can be easily adjusted by tailoring the design parameters^30,31,37–39^. Furthermore, the target stop-band can be adjusted by selecting an appropriate material for the metasurface and altering its design parameters^28,29^. In our design, silicon (Si) is used for a high refractive index since it works as a lossless dielectric material within the NIR to long-wave infrared (LWIR) range. The bottom Au mirror works as a broadband reflector from NIR to LWIR as well as an absorber. The space between the two mirrors is an empty cavity where an analyte would be placed, thereby changing the optical path length. The primary difference between *Type A* and *Type B* is the presence of nanoscale slits and whether the metasurface is supported by a handle layer or not. The two types of the MAFP structure can be fabricated by following the fabrication procedure in Supplementary Information A.

For gaseous analytes, an optical RI sensor structure of *Type A* as shown in Fig. 1a,b can be used. This consists of a top metasurface, an air cavity, and a bottom Au mirror. The top metasurface is a grating that consists of high-index Si and low-index air bars. The structural parameters of a metasurface are grating period (Λ), Si grating width (*w*), grating thickness (*t*_g_). Air cavity thickness (*t*_c_) and bottom Au thickness (*t*_Au_) are also the structural parameters but are not relevant to the metasurface itself. The top metasurface allows gas to diffuse into the air cavity through the air spaces of a width of (Λ-*w*) after which the incident region, the air space in the metasurface, and the cavity region are all filled with gas. By maximizing the influence of RI change in the sensor and detecting the small RI change caused by gas, *Type A* is well suited for gas sensing applications.

During the detection of liquid analytes, however, the suspended Si metasurface in air can be deformed or broken by unexpected mechanical pressure such as solid–liquid surface tension. Accordingly, a practical optical RI sensor of *Type B* is suggested as shown in Fig. 1c,d. The metasurface is backed by a rigid glass substrate with the thickness of *t*_h_. In addition, the low index grating bars of the metasurface are SiO_2_, as opposed to air, as air could result in a non-uniform distribution of liquid due to the presence of captured air pockets and incorrect reflectance information compared to what is expected. As a result, the proposed *Type B* structure allows for liquid to fill the cavity, and precision changes to the resonance wavelength to be made.

## Numerical and theoretical analysis

**Figure 2:**
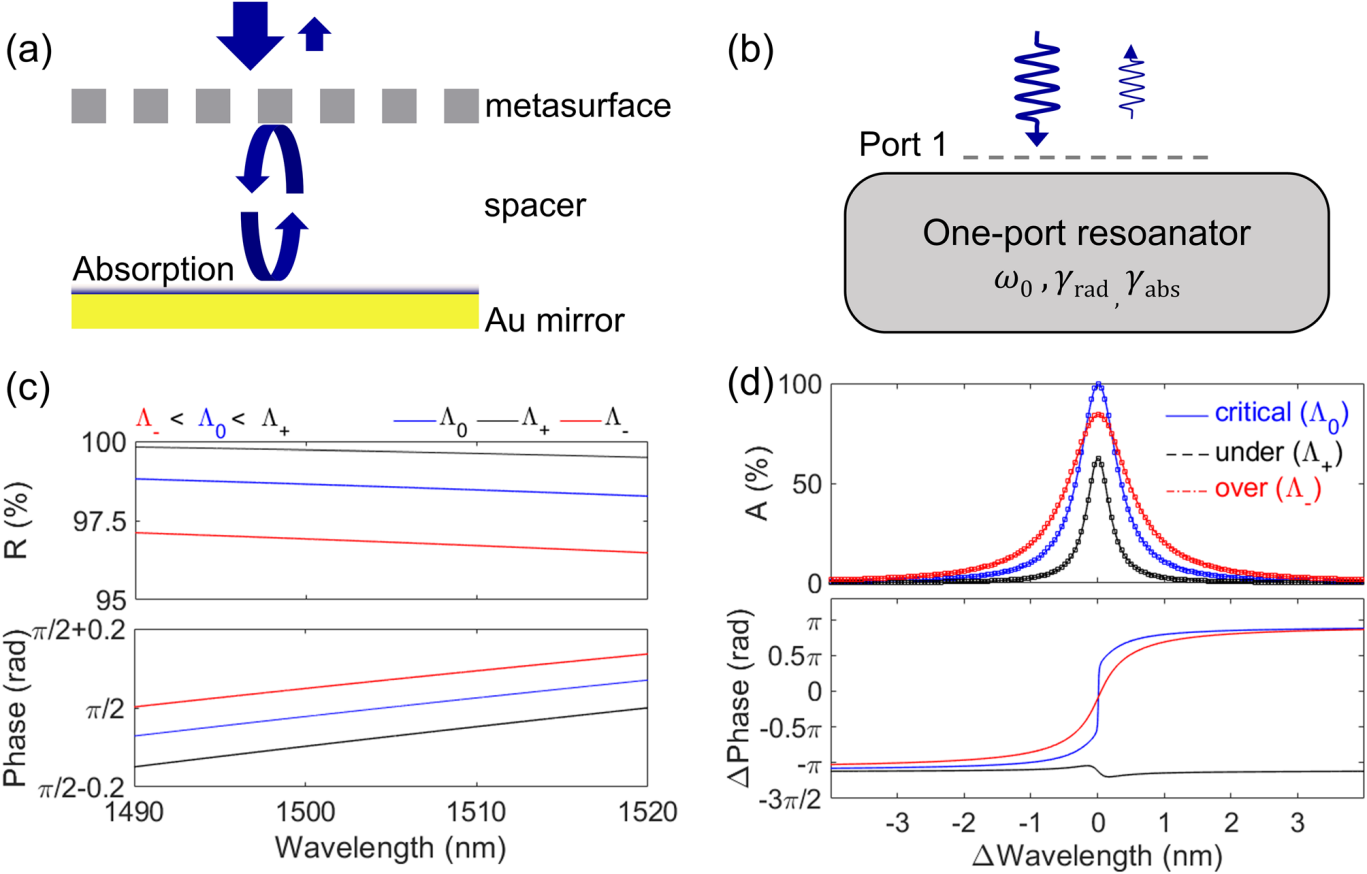
Schematic illustrations of the resonance mechanism in the metasurface-Au Fabry–Perot cavity (**a**) with a physical resonance structure and (**b**) with a one-port resonator model. (**c**) The numerical results of reflectance (upper) and phase (lower) spectra of the dielectric metasurface with three different periods (Λ_−_, Λ_0_, and Λ_+_) but with other design parameters kept constant. (**d**) The simulated absorption and phase shift spectra of the resonance structure with the three different period of the metasurface. The overlaid empty squares on each line of the absorption spectra are the result of analysis by TCMT.

To calculate the absorption and reflectance of a MAFP cavity structures, an *in-house* rigorous-coupled wave analysis (RCWA) method is utilized^40^. In addition, to understand their physical behaviors of the MAFP structure, the TCMT is employed^24,41^. Figure 2a shows a schematic view of the working mechanism using a *Type A* structure with TE-polarized incident light, resonant light, and reflected light. Unless otherwise noted, the TE-polarized light is incident on the MAFP with a normal angle. In principle, the incident light during the resonance process is absorbed in the Au layer. Since the Au layer is assumed to be thick enough, there is no light to pass through. This feature allows the MAFP cavity structure to be modeled as a one-port resonator. Therefore, the absorption (A) of a MAFP cavity is calculated as A = 1 – R – T, where R and T are reflectance and transmittance, because the total power should be conserved. Here, for the MAFP cavity, T is assumed to be zero. To understand the relationship between radiation coupling rate (*γ*_rad_) and the absorption coupling rate (*γ*_abs_) at resonance wavelength (*ω*_0_) in the MAFP, a one-port model is used as shown in Fig. 2b. First, the reflectance spectrum for three representative dielectric metasurfaces with a Λ_−_ of 976 nm, a Λ_0_ of 996 nm, and a Λ_+_ of 1016 nm suspended in air is numerically calculated. The duty cycle (DC = *w*/Λ) is fixed to 0.39, and the thickness (*t*_g_) of all three metasurfaces is 220 nm. Within the wavelength range from 1490 to 1520 nm, each metasurface of Λ_+_, Λ_0_, and Λ_−_ possesses the reflectance in the range of 99.49–99.81%, 98.27–98.82%, and 96.48–97.11%, respectively as shown in the top panel of Fig. 2c. For TM-polarized incident light, the reflectance of the metasurfaces with a Λ_0_ is less than 5% (see Supplementary Information B). The phase spectra for the three metasurfaces are shown in the bottom panel of Fig. 2c. Within the wavelength range, reflectance and phase changes in a nearly linear fashion. Using these three dielectric metasurfaces, three MAFP resonance structures are formed using the Au layer with *t*_Au_ of 100 nm on a glass substrate. The cavity thickness of the three MAFP structures is the same as 6540 nm. The associated absorption and phase spectra are shown in Fig. 2d. The MAFP cavity with the Λ_0_ shows the highest absorption of ~ 99.74% and an abrupt phase change of ~ *π*. The absorption and phase conditions indicate that the MAFP is under the critical coupling condition^38,41^. Additionally, as the incidence angle of TE-polarized light increases, the peak absorption of the MAFP gradually decreases and the corresponding dip wavelength is blue-shifted (see Supplementary Information C). The other two MAFPs show lower peak absorptions. The MAFP with Λ_+_ shows a peak absorption of 62.59%, and the phase slightly increases before the resonance wavelength and then decreases after the resonance wavelength. The phase spectra near the resonance wavelength show a unique property of an under-coupling condition^41^. The MAFP with Λ_−_ shows a peak absorption of 84.41% and a gradual increase in phase around the resonance wavelength indicating that it is under an over-coupling condition. Based on the absorption and phase spectra, the coupling condition of the MAFP structures is determined. To confirm the coupling condition of the three cases, radiation coupling and absorption coupling rates are calculated using the following absorption formula for a one-port system^24,41^,

1 $${\text{A}} = \frac{{4\gamma_{{{\text{abs}}}} \gamma_{{{\text{rad}}}} }}{{\left( {\omega - \omega_{o} } \right)^{2} + \gamma_{{{\text{tot}}}}^{2} }}$$

where *ω* is the angular frequency, *ω*_0_ is the resonant angular frequency, and *γ*_tot_ is the sum of *γ*_abs_ and *γ*_rad_. Using Eq. (1), absorption is calculated using the two coupling rates of *γ*_abs_ and *γ*_rad_, and plotted with squares on top of the absorption spectra obtained from the RCWA results as shown in Fig. 2d. The calculated absorption spectra are well fitted with the numerical results. The total coupling rates (*γ*_tot_ = *γ*_rad_ + *γ*_abs_) for critical-, over-, and under-coupled MAFPs are 0.285, 0.463, and 0.173 THz, respectively. The absorption probability (*η*_abs_ = *γ*_abs_/*γ*_tot_) and radiative probability (*η*_rad_ = *γ*_rad_/*γ*_tot_) are 0.5 and 0.5 for the critical coupled MAFP, 0.696 and 0.304 for the over coupled MAFP, and 0.195 and 0.805 for the under coupled MAFP. For the MAFP cavity with the under-coupling condition, the reflectance of the metasurface is the highest among the three mirrors. In the case of the over-coupling condition, the reflectance is the lowest among the three mirrors. Therefore, the reflectance of the metasurface should be designed to keep *η*_rad_ and *η*_abs_ identical by tailoring the metasurface parameters. As highlighted in the three examined cases, the coupling condition for the MAFP cavity can be tailored by adjusting the design parameters of the metasurface. To achieve 100% absorption at the resonance wavelength and possess high reflectance at the off-resonance wavelength range, the one-port MAFP cavity structure is advantageous since it has only one radiative leakage path to be controlled. Furthermore, as soon as the wavelength deviates from the resonance wavelength for all three cases, the absorption drops to ~ 0% and the reflectance approaches ~ 100% as shown in Fig. 2d. The high reflectance at the wavelength range of off-resonance is necessary to achieve a high SC. Accordingly, the bottom Au mirror should be thick enough to block light transmission to the exit region, in which case the top metasurface only needs to be designed to meet the critical coupling condition. The lossless dielectric metasurface is an appropriate selection for use in absorption enhanced MAFP structures, as it can achieve the necessary reflectance without material loss. Near perfect absorption can be achieved in the MAFP cavity by adjusting the radiative rate through the top dielectric metasurface and the absorption rate in the bottom Au mirror^24,38,39,41,42^. As a result, the proposed platform can be used for gases and liquids sensing by interrogating the shift in a peak or dip position along an optical spectrum with a high SC.

## Gas sensors

**Figure 3:**
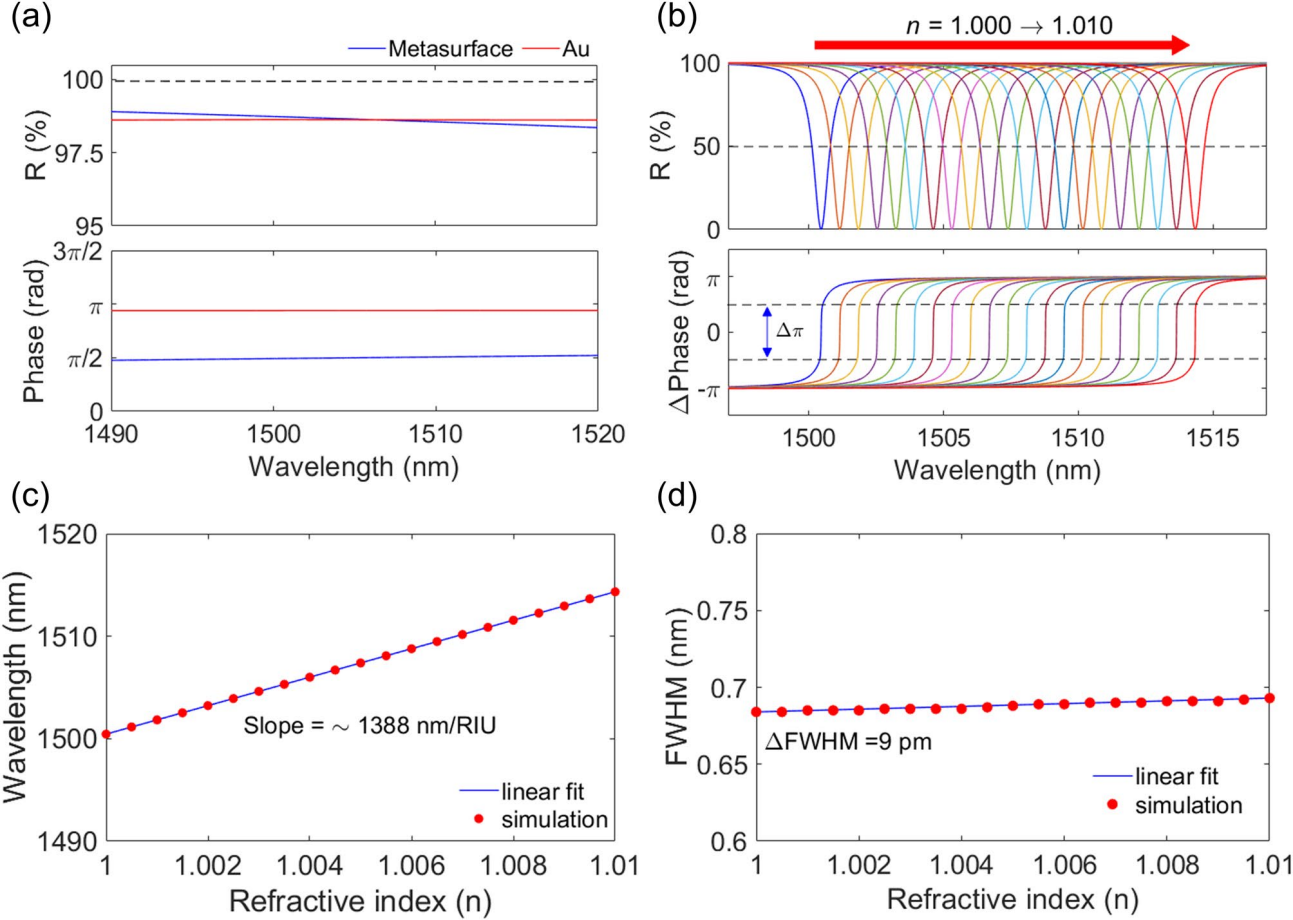
(**a**) Reflectance and phase spectra of the metasurface and Au mirror of a gas MAFP. (**b**) The reflectance and phase change spectra of the gas MAFP cavity with varying the refractive index of gaseous analyte from 1.000 to 1.010 in steps of 5 × 10^−4^. (**c**) The extracted dip position (red circles) of the gas MAFP for each refractive index, and the estimated slope (blue solid line) of ~ 1388 nm/RIU. (**d**) The extracted FWHM (red dots) and the linear fit (blue solid line). The change of FWHM within the refractive index range is 9 pm. All the results are obtained from numerical simulation.

For gaseous analytes detection, *Type A* sensor is employed. This is advantageous as compared to a metasurface having no passage other than its surface, in which the crust of the metasurface is exposed to the gases only and senses the refractive index change. The refractive indices of Si (n_Si_ = 3.48), SiO_2_ ($\text{n}_{{\text{Sio}}_{2}}$ = 1.48), air (n_air_ = 1.00), and gas are assumed to be constant within the wavelength range, but the dispersion of Au is considered^43^. For the metasurface mirror, the incident and exit region are assumed as air. The design parameters of the metasurface are a Λ of 996 nm, a DC of 0.39, and a *t*_g_ of 220 nm. The metasurface is designed to work as an appropriate high reflectance mirror as discussed in the previous section. The thickness of the metasurface is chosen to be within a two-mode regime and to work as a broadband mirror^26^. The design can achieve nearly 100% reflectance, but for absorption enhanced MAFP cavities, the reflectance is slightly lowered by tailoring Λ and/or DC keeping *t*_g_ constant. The thickness of the top dielectric metasurface is fixed for TE polarized light. Otherwise, the design excites more- or less-guided modes which would prevent it from working as a broadband high-reflectance mirror^26^. For the Au mirror, the incident medium is set as air, and the exit medium is assumed to be SiO_2_. The thickness of the Au layer *t*_Au_ is 100 nm, which is thick enough to prevent light transmission in the wavelength range. The reflectance and phase spectra of the two mirrors are numerically calculated as shown in Fig. 3a. The reflectance of the two mirrors within the wavelength range is ~ 98%. The phase shift is ~ *π*/2 at the metasurface and ~ 0.94*π* at the Au layer. To detect a minuscule change in the RI of gas, a cavity thickness *t*_cav_ of 6540 nm is chosen and the MAFP cavity can achieve enough resonance wavelength shift. When the two mirrors form an FP cavity, their reflectance and the absorption of the Au mirror together determine the Q-factor of the FP cavity and resonant absorption.

Figure 3b shows the reflectance spectra and the corresponding phase spectra as the RI of gas changed from 1.000 to 1.010 in steps of 5 × 10^−4^^19^. Since the reflectance of both mirrors is quite high, the MAFP shows a narrow enough FWHM. In addition, with the change in the RI of 5 × 10^−4^, each dip positions are well-separated with a dip-to-dip spacing of ~ 0.7 nm and without overlap below a reflectance of ~ 50% (gray dotted line). As the RI increases, the dip position is red-shifted in wavelength accordingly. All spectra show ~ 0% reflectance at each resonance wavelength and the MAFP cavity is around the critical coupling condition within the RI range. When the wavelength is away from each resonance wavelength, the reflectance approaches to ~ 100% reflectance. The phase change of *π* around the resonant absorption shows the same trend of the phase change when the critical coupling condition is met^28,31^. Moreover, the background reflectance is ~ 100% within the wavelength range. Therefore, the SC of the MAFP sensor is close to the ideal value of 100%. The dip position at the corresponding RI is extracted and plotted as shown in Fig. 3c. The red dots are the extracted values, and the blue solid line is the result of the linear fit. The wavelength shift is nearly linear with a slope of ~ 1388 nm/RIU. With a change of RI of 5 × 10^−4^, the dip wavelength shift is ~ 0.6945 nm on average. The FWHMs of the reflectance spectra of the MAFP cavity is calculated as shown in Fig. 3d. The average FWHM was 0.688 nm, and the narrow FWHM is due to the fact that MAFP possesses a high Q of ~ 2190. Since the FWHM is lower than 0.7 nm, each spectrum begins to overlap next to each other occurs when the reflectance level is above 50% as shown in Fig. 3b. Therefore, it is easy to distinguish individual dip positions. Furthermore, the total change in FWHM was only ~ 9 pm within the change of the RI. With a thicker cavity, *Type A* sensor can distinguish H_2_, O_2_, and CO_2_ without overlap below a reflectance of ~ 50% (see Supplementary Information D). In addition, to estimate the effect of fabrication imperfection on absorption, a rigorous statistical analysis is conducted (see Supplementary Information E). If the cavity thickness and DC are within tolerance, then the distribution of peak absorptions shows that the minimum peak absorption is ~ 98%.

**Figure 4:**
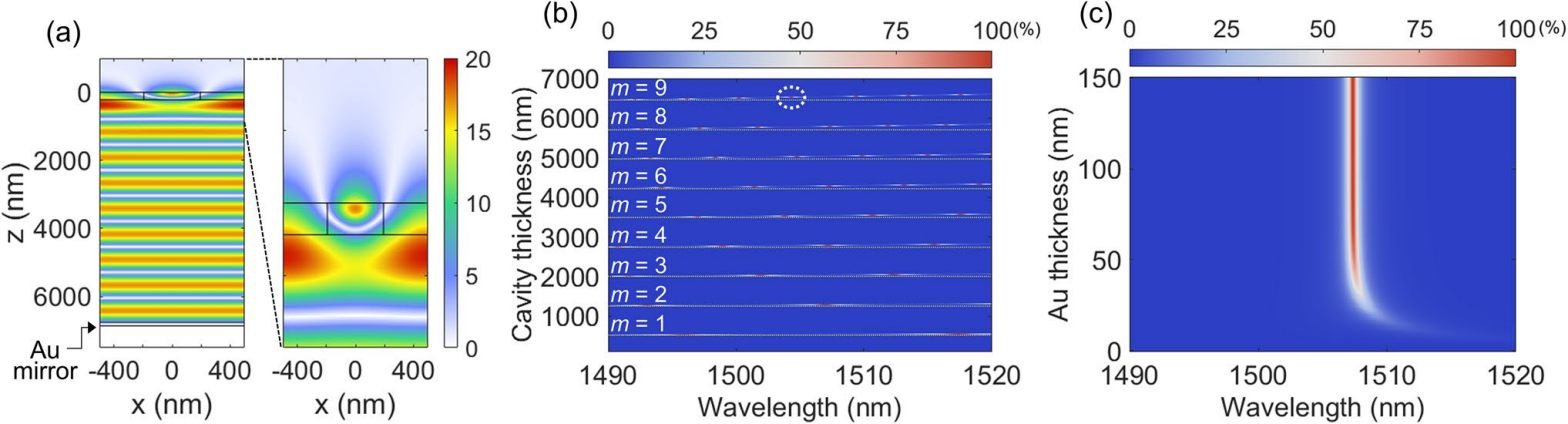
(**a**) |E_*y*_| field profile of the gas sensor filled with a gas of n = 1.005 excited at the resonance wavelength of 1507.39 nm. The enlarged image on the right shows the field profile near the metasurface. The black solid lines show the structure boundaries of the metasurface and the Au mirror. (**b**) Numerical result of absorption contour map as a function of wavelength and cavity thickness. The *m* indicates the mode number of the gas sensor. A representative mode with *m* = 9 around the critical coupling condition is indicated with a white dashed circle. Yellow dash lines represent parallel lines across the wavelength range. (**c**) Numerical result of absorption contour map as a function of wavelength and Au thickness.

Next, the effect of cavity thickness (*t*_cav_) is investigated. The |E_y_| field profile of the gas MAFP cavity filled with a gas of an RI of 1.005 at the resonance wavelength 1507.39 nm is shown in Fig. 4a. The resonance wavelength is determined by the phase shift in the top and bottom mirrors and the phase shift in the propagation in the cavity,

2 $$\frac{{4\pi nt_{c} }}{\lambda } + \phi_{meta} + \phi_{Au} = 2\pi m$$

where n is the RI of the cavity, *λ* is the wavelength, *ϕ*_meta_ and *ϕ*_Au_ are the phase shift by the metasurface and Au mirror, and *m* = 1,2,3, ···. The black solid lines indicate the outline of the metasurface and Au mirror. The excited mode inside the MAFP cavity shows the 9 anti-nodes of a standing wave, which corresponds to the resonance condition of *m* = 9. Beneath the Au mirror, it is evident that no light is transmitted thus formed a one-port resonator. The enlarged image on the right shows the mode profile inside and around the metasurface in greater detail.

The other two thickness parameters of *t*_c_ and *t*_Au_ are also investigated by mapping the absorption as shown in Fig. 4b,c. It is assumed that the MAFP cavity is filled with air, i.e., n = 1. As cavity thickness increases, the corresponding modes indicated as the mode number of *m*, are excited as shown in Fig. 4b. According to Eq. (2), the difference in optical cavity thickness (n∆*t*_c_) between the nearest modes at the same wavelength is uniform. In addition, for a longer wavelength, n∆*t*_c_ becomes larger. Therefore, at the two-end wavelength of *λ*_1_ of 1490 nm and *λ*_2_ of 1520 nm, the difference of n∆*t*_c_ (n∆*t*_c,2_−n∆*t*_c,1_) is accumulated as the mode number increases as shown in Fig. 4b. Accordingly, with a longer cavity, the sensitivity of an FP-type optical cavity can be enhanced^22,23^. In the case of the Au mirror, Au thickness determines the reflectance, which in turn affects absorption and the Q-factor of the MAFP cavity. The thickness dependency of Au on the absorption is shown in Fig. 4c. As the thickness of Au increases, the absorption peak position initially shifts toward a shorter wavelength, and the corresponding absorption increases until *t*_Au_ is approximately equal to 50 nm. Above an Au thickness of ~ 50 nm, absorption and the corresponding peak wavelength remain almost constant. Below the Au thickness of 50 nm, the Au mirror starts to transmit light into the exit region, and hence the reflectance of the Au mirror decreases. Absorption is, therefore, reduced because of the decrease of the resonant absorption in the Au layer. Overall, the bottom Au mirror should be thicker than 50 nm and form a one-port resonator to achieve the critical coupling condition.

## Liquid sensors

**Figure 5:**
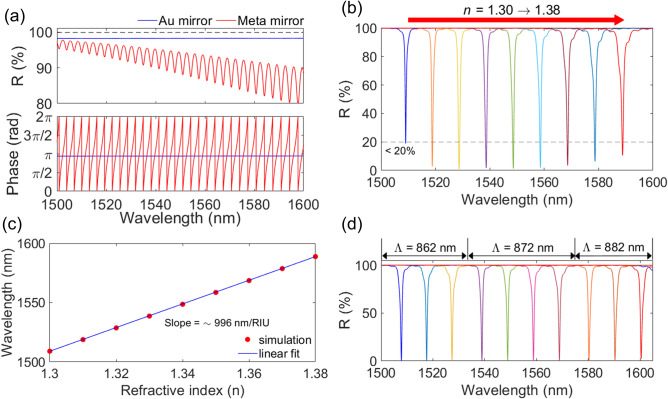
(**a**) Reflectance and phase spectra of the metasurface on a glass substrate and the bottom Au mirror. (**b**) Reflectance spectra of the liquid sensor by varying the refractive index of liquid from 1.30 to 1.38 in steps of 0.01. (**c**) The extracted dip position (red dots) of the liquid sensor for each reflectance spectrum and the estimated slope (blue solid line) of ~ 996 nm/RIU. (**d**) Reflectance spectra of the liquid sensor with three different periods of 862, 872, and 882 nm covering the refractive index range from 1.30 to 1.39 in steps of 0.01. All the results are obtained from numerical simulation.

For liquid analytes detection, a *Type B* sensor as shown in Fig. 1c,d is preferable. The reflectance and phase spectra of the two mirrors are calculated as shown in Fig. 5a. For the metasurface mirror, the incident and exit regions are assumed to be air. The design parameters of the metasurface are a Λ of 870 nm, a DC of 0.316, and a *t*_g_ of 270 nm. Since the metasurface is covered with a 250 µm-thick glass handle, the reflectance of the metasurface showed an interference pattern with a free spectral range (FSR) of 3.26 nm. In addition, the phase spectra of the metasurface showed a fast phase change. A bottom Au mirror with a *t*_Au_ of 100 nm is used, identical to that which was used in the gas MAFP cavity.

As the MAFP cavity with a *t*_c_ of 7.8125 µm is filled with a liquid varying from n = 1.30 to n = 1.38 in steps of 0.01^2,22^, the dip position is red-shifted as shown in Fig. 5b. All reflectance dips are below 20%. Due to the interference caused by the glass substrate, the reflectance spectra show the overlap between the interference pattern and the resonance. As the RI of the liquid increases, the coupling condition changes from the under-coupling, the critical coupling, finally to the over-coupling. As a result, the FWHM became gradually larger, and the reflectance dip position changed in accordance with each coupling condition. When the RI is lower than 1.32, the MAFP cavity is in the under-coupling condition. As the RI exceeded 1.34, the MAFP cavity entered the over-coupling condition. In between the two RI boundaries, the MAFP cavity is around the critical coupling condition. Even though the thick glass handle introduces ripples in reflectance spectra, the dip wavelength changes almost linearly with the slope of ~ 996 nm/RIU as shown in Fig. 5c. The red circles indicate the extracted dip wavelength from Fig. 5b, and the blue solid line is the linear fit of the result. Since an RI change of 0.08 is large enough to change the coupling condition of the MAFP cavity, the period of the metasurface is adjusted to increase absorption and SC as shown in Fig. 5d. From n = 1.30 to n = 1.32, the metasurface with a Λ of 862 nm meets a near-critical coupling condition as it showed ~ 100% absorption. As Λ becomes 872 nm, the metasurface achieves a near-critical coupling conditions within an RI range from 1.33 to 1.36. Next, Λ is increased to 882 nm to cover the RI between 1.37 and 1.39. It is noteworthy that only Λ is tailored while others are fixed to achieve near-perfect absorption. Ultimately, the absorption enhanced MAFP cavity can be further optimized by tailoring other parameters of the metasurface. Considering the narrow FWHM and the SC level, the *Type B* structure is competitive compared to other sensor structures (see Supplementary Information F).

**Figure 6:**
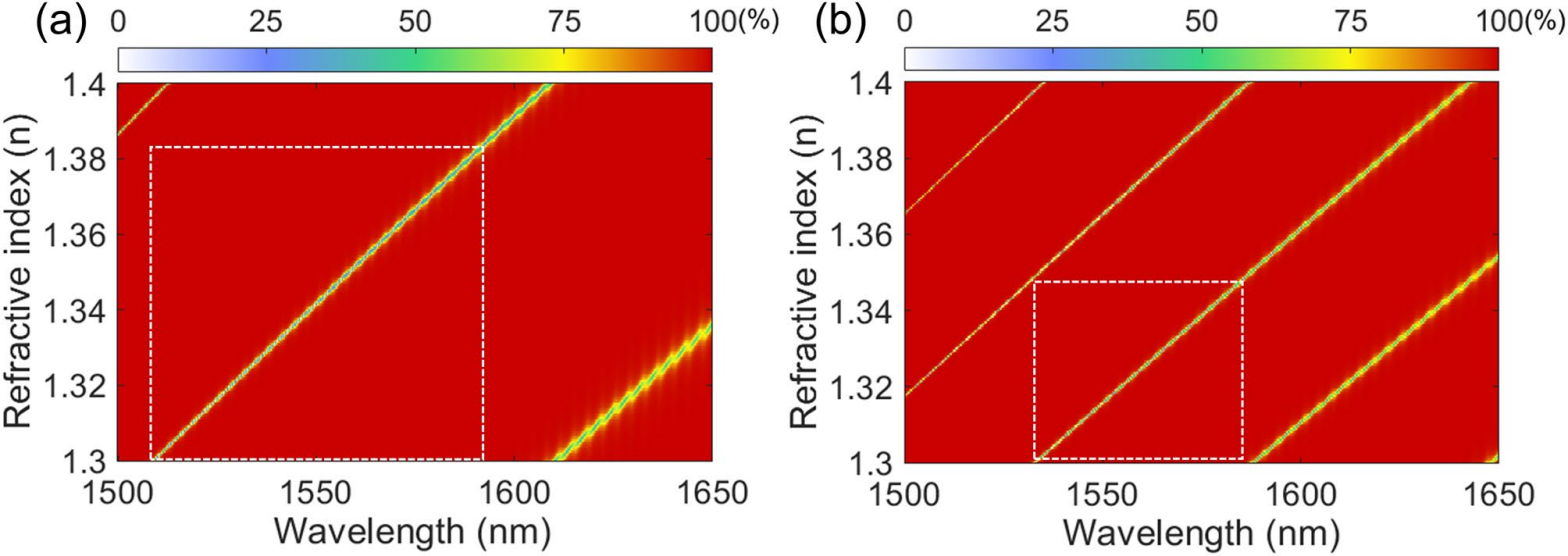
Calculated reflectance contour map as a function of wavelength and refractive index for a cavity thickness of (**a**) 7.8125 µm and (**b**) 15.6250 µm. The white-dash box indicates the region where only one resonance mode can be found within the wavelength range as the refractive index increases.

The effect of cavity thickness of the liquid MAFP cavity on the single-mode operation is investigated by mapping reflectance as a function of wavelength and RI as shown in Fig. 6. Since RI and the wavelength range are set wider for liquid sensing than for gas sensing, there can be more modes excited within the wavelength range. Two MAFP cavities with *t*_c_ of 7.8125 µm and 15.6250 µm are investigated. All other parameters are the same as those used in Fig. 5b. In case of the shorter cavity, the reflectance contour map shows three excited modes within the simulation domain. The MAFP with the longer cavity shows four modes. The white-dash boxes indicate the region where only a single mode exists. For the short cavity, the single-mode operation is achieved when RI is varied from 1.300 to 1.385 within the wavelength range of 1509.0 nm to 1593.4 nm. The shift of the resonance wavelength is ~ 84 nm when the RI is altered by ~ 0.085. The corresponding S is ~ 993 nm/RIU. With the longer cavity as shown in Fig. 6b, there is one additional mode and the four excited modes are arranged more closely to each other. In other words, the single-mode region diminishes. The shift of the resonance wavelength is ~ 52 nm with varying RI by ~ 0.048. In this case, the S of ~ 1091 nm/RIU is achieved due to the longer cavity. Consequently, the MAFP resonator with the shorter cavity is appropriate to detect a wide range of RI. From the perspective of S, the longer cavity is better. However, RI and wavelength range gets narrower, and the sensor becomes bulky. Cavity thickness, therefore, should be decided not only considering S but also the target RI range. Q and SC should be considered as well.

## Conclusion

We proposed and analyzed one-port MAFP cavities with a near-perfect absorption at a resonance wavelength and a near-perfect reflectance at an off-resonance wavelength range. The design parameters of the top dielectric metasurface were tailored to tune *γ*_rad_ and *γ*_abs_ of the one-port MAFP cavity, which were backed with a 100 nm thick Au mirror. The coupling condition of the MAFP cavity was first investigated using TCMT, and the dielectric metasurface was then adjusted to meet the critical coupling condition. Accordingly, MAFP cavities for gas and liquid sensing were investigated. The gas MAFP showed an S of ~ 1388 nm/RIU and an FWHM of less than 0.7 nm. It could resolve the RI of 5 × 10^−4^ with an SC of ~ 100%. The liquid MAFP showed an S of ~ 996 nm/RIU when the RI changed from 1.30 to 1.38. By tuning the period of the metasurface but keeping its thickness, an SC of ~ 100% was achieved for each specific RI range. Furthermore, the effect of the cavity thickness of the MAFP on a single-mode operation indicated that a wider RI range can be analyzed with a thinner cavity, but with a thicker one, a larger S can be achieved.

## Supplementary Information

Supplementary Information.
