# Supplementary material for: Critically coupled Fabry–Perot cavity with high signal contrast for refractive index sensing
Source: Sci Rep. 2021 Oct 1;11:19575. doi: 10.1038/s41598-021-98654-w (PMC8486813; doi:10.1038/s41598-021-98654-w)
Supplement: Supplementary file 1 — Supplementary Information. [file 41598_2021_98654_MOESM1_ESM.pdf]

Supplementary Information

Critically coupled Fabry-Perot cavity with high signal contrast for refractive index sensing

Gyeong Cheol Park[1, *] and Kwangwook Park[2, 3, 4, *]

[1]Electronics and Telecommunications Research Institute, Daejeon 34129, Republic of Korea
[2]Division of Advanced Materials Engineering, Jeonbuk National University, Jeonju 54896, Republic of Korea
[3]Hydrogen and Fuel Cell Research Center, Jeonbuk National University, Jeonju 54896, Republic of Korea
[4]Department of Energy Storage/Conversion Engineering of Graduate School, Jeonbuk National University, Jeonju 54896, Republic of Korea
[*]E-mail: gcpark@etri.re.kr (GCP), kwangwook.park@jbnu.ac.kr (KP)

A. Fabrication procedure

The fabrication of the proposed MAFP cavity is feasible, and it can be fabricated following the procedure as shown in Fig. S1.

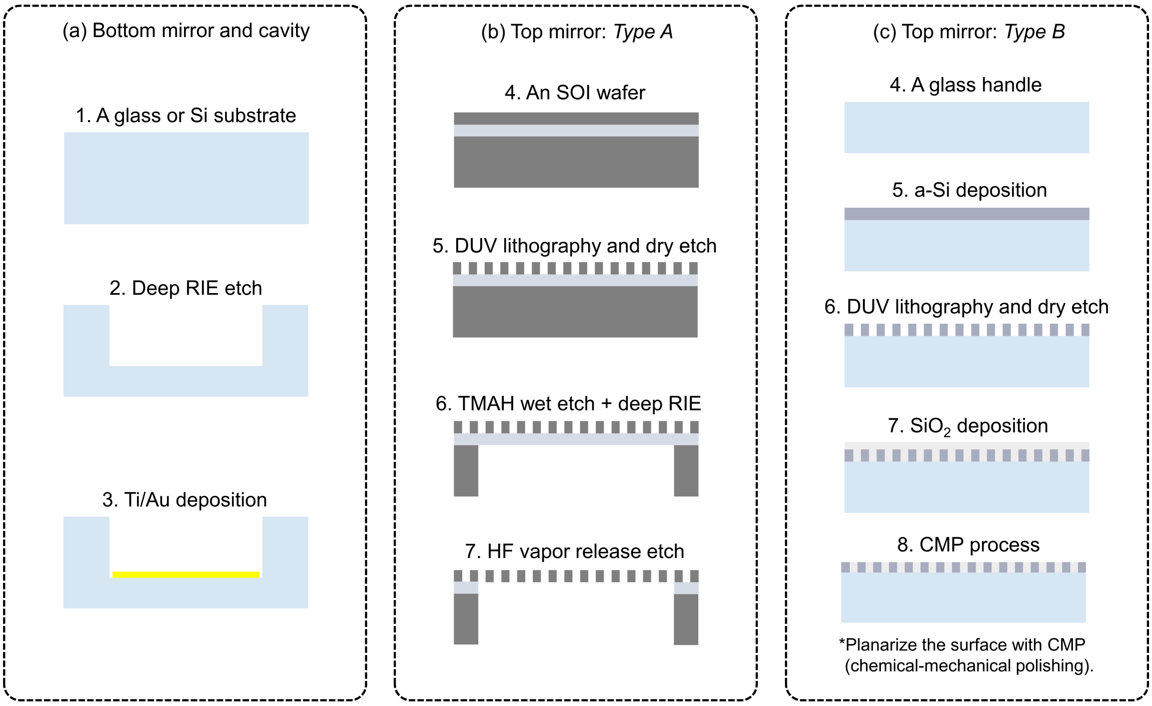

Figure S1. Schematic illustration of the fabrication procedure for (a, b) *Type A* structure and for (a, c) *Type B* structure.

B. Polarization-dependent reflectance of the metasurface

The metasurface of the MAFP cavity is a one-dimensional grating structure and designed for TE-polarized light. Thus, it is sensitive to the polarization state of incident light. The reflectance spectra of the metasurface for TE- and TM-polarization are shown in Fig. S2. The design parameters of the metasurface for *Type A* are used for comparison. For the TE-polarized light, the top metasurface presents high reflectance, and hence it can possess perfect absorption and high signal contrast (SC) as discussed in Fig. 3(b). However, the metasurface presents low reflectance for TM-polarized light[1,2]. Therefore, for TM-polarized light, it cannot achieve high absorption.

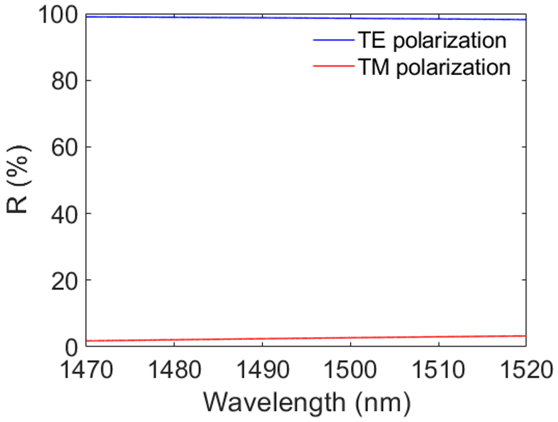

Figure S2. The numerical result of reflectance spectra of the dielectric metasurface for TE- and TM-polarized incident light. TE-polarization represents E_y field of incident light is parallel to the grating bar, and TM-polarization represents H_y field of incident light is parallel to the grating bar.

C. Angle-dependent absorption of the *Type A* MAFP cavity

The reason for achieving the critical coupling condition in this system is to possess a high SC. At the critical coupling condition, the reflectance drops to zero. Although the system slightly deviates from the critical coupling condition, the resonant absorption is not immediately disappeared but slightly decreased as shown in Fig. S3. On top of that, the MAFP cavity shows ~100% reflectance at the off-resonance wavelength region under oblique incidence. The MAFP cavity shows a descent level of SC.

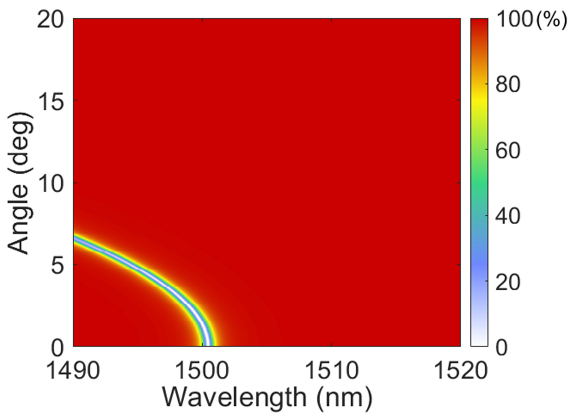

Figure S3. The numerical result of a reflectance map of the dielectric metasurface as a function of wavelength and incidence angle.

Within the simulation domain, the dip position is blue-shifted as the incidence angle increases. The maximum dip level is ~15% at the wavelength of 1490 nm. At normal incidence, the dip level is close to 0% at 1500.45 nm. When the incidence angle is 1°, the dip level is 0.16% at 1500.45 nm.

D. *Type A* MAFP with a longer cavity

Using *Type A* structure, the representative MAFP cavity operates under the critical coupling condition at least up to the RI change of 0.01. The MAFP cavity shows the average FWHM of 0.688 nm with less than ~9 pm variation as the RI changes from 1.000 to 1.010. With the cavity thickness of 6540 nm, it shows that it can resolve the change of 5×10^{-4} RIU. Furthermore, the criterion is the overlap in the reflectance spectrum of 50% as shown in Fig. 3(b). This criterion was set conservatively by considering the potential background noise.

To resolve the change of 1×10^{-5} RIU, a longer RI sensor such as a fiber-based RI sensor is necessary. Although, as discussed in Fig. 4(b), it can have a higher resolution and Q-factor if the proposed MAFP have a thicker cavity. If the cavity thickness of the MAFP structure increases from 6540 nm to 5×6540 nm, then it can distinguish three exemplary gases of H_2, O_2, and CO_2 which satisfies the criterion of the 50% overlap as shown in Fig. S4.

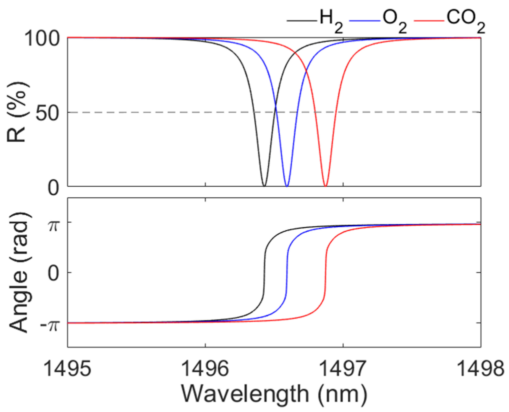

Figure S4. The numerical result of reflectance (upper) and phase (lower) spectra for three different gases of H_2, O_2 and CO_2.

The refractive indices and the corresponding dip wavelengths for three gases are summarized in Table S1. For this simulation the DC was 0.40, instead of 0.39 to achieve the critical coupling condition. Other design parameters were kept the same.

Table S1. Refractive indices of three gases and the corresponding dip wavelength.

Gas	n (at λ = 1500 nm)	Dip wavelength (nm)
H_2	1.00013620	1496.43
O_2	1.00024768	1496.60
CO_2	1.00043860	1496.88

As a reference, the recent results of on-chip gas RI sensors are summarized in Table S2, which compares sensitivity (S), wavelength range, FWHM, refractive index (RI) range, type of resonance, and signal contrast (SC). Among the on-chip based RI sensors, the proposed structure shows the descent performance in terms of sensitivity, FWHM, and SC.

Table S2. Summary of the previously reported on-chip gas sensors and the present work.

Year	Sensitivity (nm/RIU)	Wavelength range (nm)	FWHM (nm)	RI range	Type	†SC
2016[3] (N)	885	800-1100	8	1.0~1.05	SPP	1
2020[4] (N)	1052	1480-1490	0.38	1.001~1.005	GMR	0.97
2021[5] (N)	1317	1300-1450	1.74	1.00 ~ 1.10	SPP	0.9~1.0
This work (N)	1388	1490-1520	0.688	1.000~1.010	FP	1

†SCs are estimated from the data in each reference and N indicates numerical result.

E. Statistical analysis of the fabrication imperfection

Regarding the fabrication imperfection, two cases are investigated assuming that the period of a metasurface is kept the same. The first case is when metasurface thickness (t_g), DC, and cavity thickness (t_c) deviate from the set parameters. As the second case, DC and cavity thickness are considered. Prior to fabrication the silicon (Si) gratings of a metasurface, the thickness of a Si layer can be measured to eliminate the uncertainty caused by the metasurface thickness. To study the design sensitivity of a MAFP structure, the values of each parameter are randomly generated, and then the maximum absorption and its corresponding peak wavelength are calculated. The randomly generated values of the parameters follow a normal distribution (Equation. S1), where μ is the mean and σ is the standard deviation. We generate 2,000 samples, and each parameter is uncorrelated. For the first case, the design parameters are shown in Table S3.

Equation S1. Normal distribution.

$$\mathrm{f(x)} = \frac{1}{\sigma\sqrt{2\pi}} exp\left[-\frac{1}{2}\left(\frac{x-\mu}{\sigma}\right)^2\right]$$

Table S3. Simulation parameters for the case 1.

	Metasurface thickness (nm)	DC	Cavity thickness (nm)
μ	220	0.39	6540
σ	3	0.005 (4.98 nm)	5
3σ	9	0.015 (14.94 nm)	15

Figure S5(a-c) shows the normal distribution of t_g, DC, and t_c, respectively. As shown in Fig. S5(d), the lowest maximum absorption is slightly above 20% and for many cases, the absorption is lower than 90% and the peak absorption wavelengths are distributed within the wavelength range of ~10nm. When considering the signal contrast of the MAFP structure, this low absorption is not desirable.

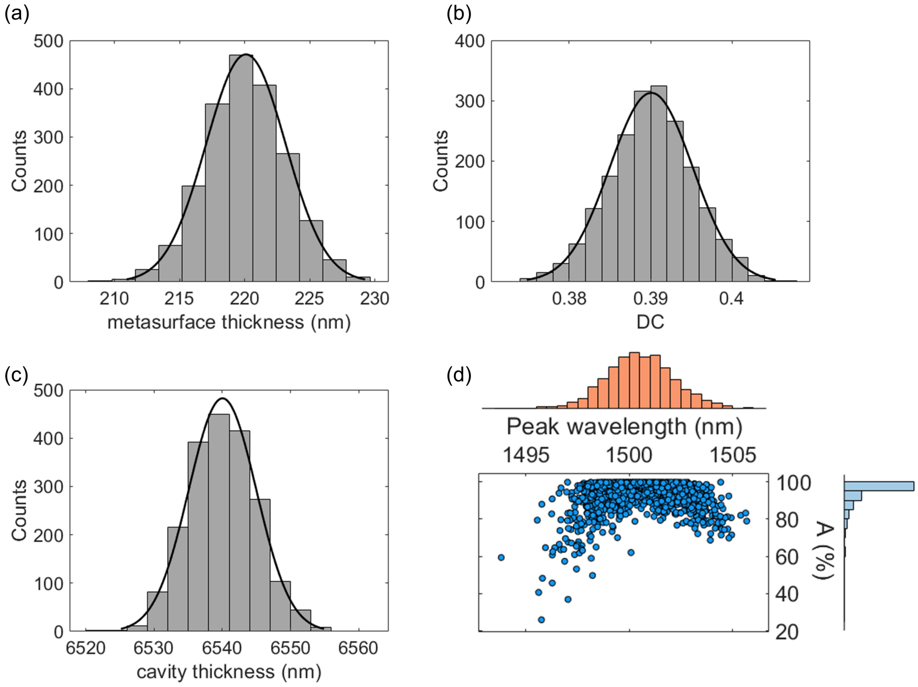

Figure S5. The distribution of peak absorptions and corresponding wavelengths caused by unwanted fabrication imperfection, by randomly generated three parameters of (a) metasurface thickness, (b) DC, and (c) cavity thickness. (d) Scatter plot of peak absorptions and corresponding wavelengths and distribution histogram for peak wavelength distribution (top inset) and for maximum absorption distribution (right inset).

In advance to the fabrication of the MAFP structure, we can measure the Si layer where the metasurface will be formed. Therefore, we can eliminate one more uncertainty, t_g, by choosing the desired Si layer or by modifying the grating design parameters. Therefore, there are two uncontrollable parameters: DC and t_g. For the second case, the design parameters are shown in Table S4.

Table S4. Simulation parameters for the case 2.

	DC	Cavity thickness (nm)
μ	0.39	6540
σ	0.005 (4.98 nm)	5
3σ	0.015 (14.94 nm)	15

Figure S6(a, b) shows the normal distributions of DC and t_g. When only two parameters are uncertain, the maximum absorption is more than 98% within the peak wavelength spread of ~7nm as shown in Fig. S6(c). Therefore, we can achieve a high SC using the MAFP cavity. The standard deviation for each parameter can be achievable by optimizing the fabrication process.

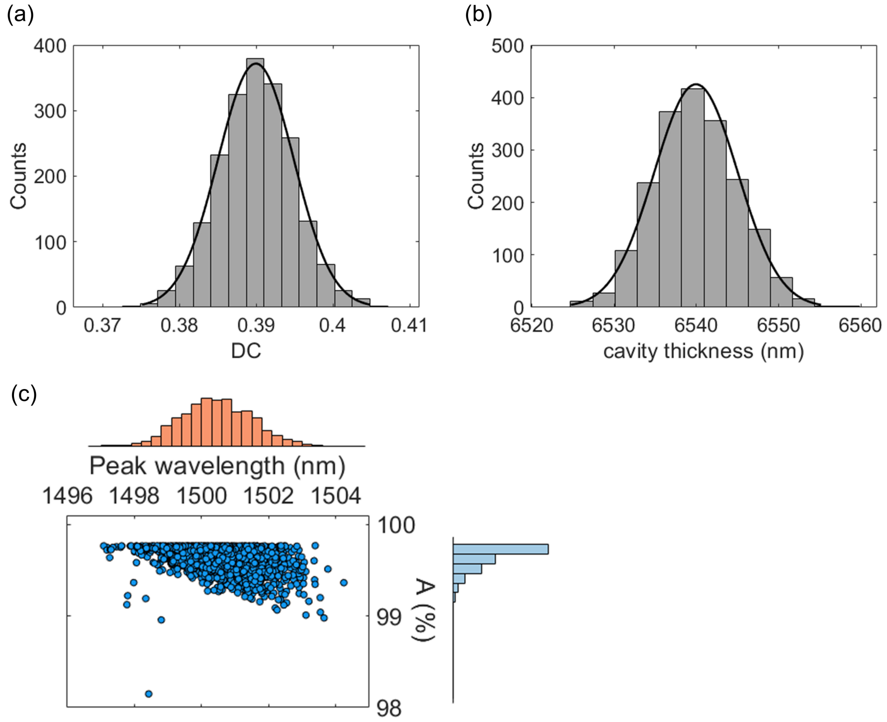

Figure S6. The distribution of peak absorptions and corresponding wavelengths caused by unwanted fabrication imperfection, by randomly generated three parameters of (a) DC and (b) cavity thickness. (c) Scatter plot of peak absorptions and corresponding wavelengths and histogram of peak wavelength distribution (top inset) and for maximum absorption distribution (right inset).

F. Summary of the recent results of liquid RI sensors

The recent results of liquid RI sensors are summarized in Table S5, which compares S, wavelength range, FWHM, RI range, type of resonance, and SC.

Table S5. Summary of the previously reported liquid sensors and the present work.

Year	Sensitivity (nm/RIU)	Wavelength range (nm)	FWHM (nm)	RI range	*Type	†SC
2015[6] (E)	~32	520-630	1~2	1.333-1.413	GMR	0.875
‡2016[7] (N)	1445	2440-2780	50.1	1.312-1.352	Plasmonic	~0.95
2017[8] (E)	137	~840	~2	1.3324-1.3444	GMR	0.8
2019[9] (E)	574	780-840	~7.2	1.3285-1.3468	SPP	-
2019[10] (N)	860	1320-1420	~2.2	1.32-1.42	TP	0.85-1.0
2020[11] (N)	137-252	-	-	-	BIC	-
2020[12] (E)	680	400-850	>100	1.33-1.38	FP	-
This work (N)	996	1500-1600	~1	1.30-1.38	FP	0.81-1.0

†SCs are estimated from the data in the references.

‡In this work, the calculated spectrum for materials with the RI difference of 0.01 shows the spectrum overlap up to the absorption level of ~0.95 because of the large FWHM. Therefore, the very narrow level of the nonoverlapped spectrum from 0.95 to the maximum level of 1 is carefully used to determine the shift of the peak of absorbance. However, in our work, there is no spectrum overlap of the calculated spectrum for materials with the same RI difference of 0.01. Therefore, it can resolve even lower RI change.

*GMR (guided mode resonance), SPP (surface plasmon polariton), TP (Tamm plasmon), FP (Fabry-Perot), and E (experimental result) / N (numerical result).

Supplementary References

1. Rao, Y. *et al*. Long-wavelength VCSEL using high-contrast grating. *IEEE J. Sel. Top. Quantum Electron.* **19**, 1701311 (2013).
2. Park, G. C., Taghizadeh, A. & Chung, I.-S. Hybrid grating reflectors: origin of ultrabroad stopband. *Appl. Phys. Lett.* **108**, 141108 (2016).
3. Yong, Z., Zhang, S., Gong, C. & He, S. Narrow band perfect absorber for maximum localized magnetic and electric field enhancement and sensing applications. *Sci. Rep.* **6**, 24063 (2016).
4. Liao, Y.-L. & Zhao, Y. Ultra-narrowband dielectric metamaterial absorber with ultra-sparse nanowire grids for sensing applications. *Sci. Rep*., **10**,1480 (2020).
5. Yan, Z. *et al*. Perfect absorption and refractive-index sensing by metasurfaces composed of cross-shaped hole arrays in metal substrate. *Nanomaterials* **11**, 63 (2021).
6. Xiao, G. *et al*. A tunable submicro-optofluidic polymer filter based on guided-mode resonance. *Nanoscale* **7**, 3429-3434 (2015).
7. Cheng, Y., Mao, X. S., Wu, C., Wu, L. & Gong, R. Infrared non-planar plasmonic perfect absorber for enhanced sensitive refractive index sensing. *Opt. Mater.* **53**, 195-200 (2016).
8. Triggs, G. J. *et al*. Chirped guided-mode resonance biosensor. *Optica* **4**, 229-234 (2017).
9. Gao, B. *et al*. Nanoscale refractive index sensors with high figure of merit via optical slot antennas. *ACS Nano*, **13**, 9131-9138 (2019).
10. Qin, L., Wu, S., Zhang, C. & Li, X. Narrowband and full-angle refractive index sensor based on a planar multilayer structure. *IEEE Sens. J.*, **19**, 2924-2930 (2019).
11. Maksimov, D. N., Gerasimov, V. S., Romano, S. & Polyutov, S. P. Refractive index sensing with optical bound states in the continuum. *Opt. Express*, **28**, 38907-38916 (2020).
12. Shapturenka, P., Stute, H., Zakaria, N. I., DenBaars, S. P. & Gordon, M. J. Color-changing refractive index sensor based on Fano-resonant filtering of optical modes in a porous dielectric Fabry-Pérot microcavity. *Opt. Express* **28**, 28226-28233 (2020).
